# Broadband Bi-Directional All-Dielectric Transparent Metamaterial Absorber

**DOI:** 10.3390/nano12234124

**Published:** 2022-11-22

**Authors:** Miao Cao, Xiaojun Huang, Lina Gao, Xiaoyan Li, Linyan Guo, Helin Yang

**Affiliations:** 1 College of Communication and Information Engineering, Xi’an University of Science and Technology, Xi’an 710054, China; 2 College of Physical Science and Technology, Northwestern Polytechnical University, Xi’an 710129, China; 3 School of Geophysics and Information Technology, China University of Geosciences, Beijing 100083, China; 4 College of Physical Science and Technology, Central China Normal University, Wuhan 430079, China

**Keywords:** bi-direction, all-dielectric, transparency, polarization insensitivity, angle stability

## Abstract

Water-based absorbers have shown great development potential in the past few years. In this paper, an all-dielectric transparent bi-directional water-based broadband metamaterial absorber is designed. The simulation results indicate that absorptance of the absorber is over 90% in 5.7–41.6 GHz, and its fraction bandwidth is 151.8%. The experimental results are greatly consistent with the simulations. The designed absorber has excellent performances of polarization insensitivity, oblique incidence stability and thermal stability. When the absorptance is more than 0.8, the maximum incident angle reaches 40° in TE mode and is over 60° in TM mode. In 0–80 °C, absorptance of the absorber is hardly changed. Because of the optical transparency of the designed absorber, it can be extensively used in stealth window weapons and electromagnetic compatibility equipment.

## 1. Introduction

The origins of metamaterial absorbers can be traced back to 2002, when Engheta first proposed absorbing screens based on metamaterial surfaces [1]. Landy designed the perfect metamaterial absorber (MA) in 2008 [2], and since then, MAs have attracted wide attention because of their superior ability to absorb electromagnetic (EM) waves [3,4]. However, a more attractive advantage of MAs is their thinner thickness. MAs have been widely studied in the fields of EM stealth [5,6], sensing [7], energy transmission [8] and so on [9,10,11,12]. According to the absorption frequency band, MAs can be divided into three categories: single-frequency MAs [13], multi-frequency MAs [14,15] and broadband MAs [16,17,18]; depending on the type of aggregate element loaded, they are divided into active [12] and passive MAs [19]; there are also special MAs, such as reconfigurable absorbers based on graphene, phase change materials [20,21] and toroidal dipole absorbers [22]. Due to the development of absorbing materials and the expansion of application scenarios, broadband absorbing materials have been favored by researchers [23,24,25,26].

Water is the most common substance in nature. Because of its high dielectric constant caused by its dispersion characteristics, it has displayed a great potential in broadband absorbers [27,28,29,30]. In 2015, inspired by Rybin, Popa and Cummer, Andryieuskiet et al. certificated the possibility of utilizing the dynamic characteristics of water in microwave all-dielectric metamaterial [31,32,33] for the first time. After that, the water-based (WB) all-dielectric metamaterial absorber showed its unique charm in broadband absorption [34,35,36]. At the same time, Mikhail Odit et al. designed an adjustable WB metamaterial absorber by using the fluidity of water [37]. Zhao et al. used the broadband microwave absorption technology realized by the WB metamaterial structure and introduced water into the MA structure unit as the main resonance element. For the water drop structure, over 90% microwave absorptivity was realized at 7.5–15 GHz, while for the water pipe structure, over 90% microwave absorptivity was achieved in 5–15 GHz [38]. The same year, Shen et al. proposed transparent MA injected water, which can not only realize broadband microwave absorptivity but also tuneable infrared radiation. The absorber can absorb more than 90% broadband microwaves in 6.4–23.7 GHz [39]. Xie et al. proposed an all-dielectric MA, which was made of a sub-wavelength water-based resonator without metal grounding. The theoretical and experimental data indicated that the MA had highly uniform ideal absorptivity at 7.74–23.56 GHz and ideal thermal stability at 0–100 °C [40]. Wang et al. introduced an all-dielectric WB absorber. The absorptance of the MA was over 90% at 10.45–11.20 GHz, and it was equal to a fraction bandwidth of 6.9% [41]. Lu et al. proposed an all-dielectric WB transparent absorptive material. The simulation indicated that the absorptance of the WB absorber was over 90% at 7.28–28.22 GHz, which also had great robustness of the thermal and oblique incident angle [42]. Based on the development of transparent water-based metamaterials, most water-based absorbers pursue wideband absorption in the microwave band, but the bandwidth of these absorbers is not wide enough and uni-directional, which greatly limits their application scenarios.

In pursuit of wider bandwidths and expanding absorption directions, in this paper, based on the mixed medium of flower-shaped water layer and resin, an all-dielectric transparent bi-directional broadband water-based MA is proposed. The flower-shaped structures in the +*z* and −*z* directions are mirror symmetry. Simulations indicate that the EM absorptance of the WB absorber exceeds 0.9 in 5.7–41.6 GHz with a fraction bandwidth of 151.8%. For polarization insensitivity, the absorptance of the absorber is unchanged with the polarization angle changing at 0–45°. Furthermore, for oblique incidence stability, in the TE mode, the absorptance is more than 0.8 with the incident angle increasing at 0–40°, and in the TM mode, the absorptance is more than 0.9 as the incident angle increases from 0° to 60°. The angular stability of the absorber is verified by experiments. As for thermal stability, the absorptance of the WB absorber is hardly changed in the temperature range of 0–80 °C. Based on the above superior performance and the advantage of transparency, this absorber has great application potential in stealth window weapons and electromagnetic compatibility equipment.

## 2. Materials and Methods

**Figure 1:**
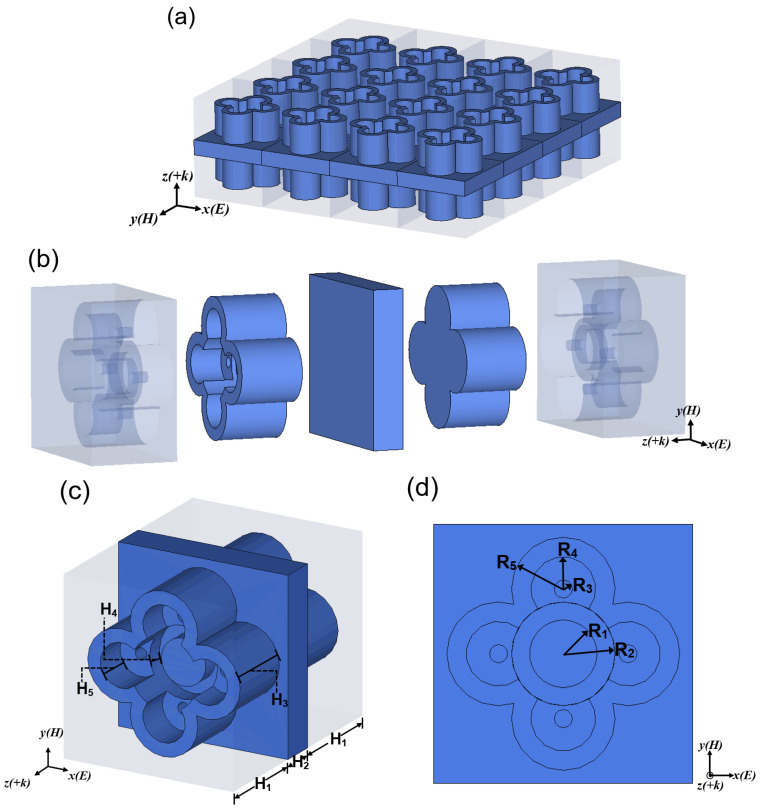
(**a**) Schematic of the MA; (**b**) structure profiling diagram; (**c**) side view; (**d**) positive view.

As illustrated in Figure 1a,b, there are five layers in the structure unit of the MA: resin layer, flower-shaped water layer, full water layer, flower-shaped water layer and resin layer. The permittivity of the resin is 3.0, and the tangent loss is 0.001; the permittivity of water follows the Debye equation in Equation (1). In Figure 1c, the height of the resin layer, full water layer and flower-shaped water layer are H_1_, H_2_ and H_3_, respectively. The flower-shaped water layer has four round petals and a cylindrical flower center, and each petal has a round terrace with round holes and the height of the flower center and the round terrace is H_4_. The height of the round terrace from the petal edge is H_5_. From Figure 1d, there are five different circles in the flower and their radii are R_1_, R_2_, R_3_, R_4_ and R_5_. The final structural dimensions of the unit structure are displayed in Table 1.

We use EM simulation software (CST Studio Suite) to study the absorptive performance of the bi-directional WB absorber. In the simulation process, the dielectric constant of water at the microwave frequency is introduced as the Debye equation [33]:

(1) $$\varepsilon (\omega ,T_{water})=\varepsilon_{\infty }(T_{water})+\frac{\varepsilon_{0}(T_{water})-\varepsilon_{\infty }(T_{water})}{1-i\omega \tau (T_{water})}$$

in which, *ε*_∞_, *ε*_0_ and *τ* represent high-frequency dielectric constant, static dielectric constant and relaxation time, respectively. This dependence of water on temperature makes it possible to use temperature as a mean to adjust the EM characteristics of water-based objects, or to maintain stable EM characteristics at different temperatures, which is worth exploring. The fitting technique is used to fit the results of the dielectric constant with frequency. In CST, unit cell boundary conditions are applied in *x* and *y* directions, and open boundary conditions are used in the z direction. The propagation of EM waves is along the z axis. In simulation, the frequency solver is applied. The mesh type and size are set to tetrahedral and adaptive, respectively.

## 3. Results and Discussion

**Figure 2:**
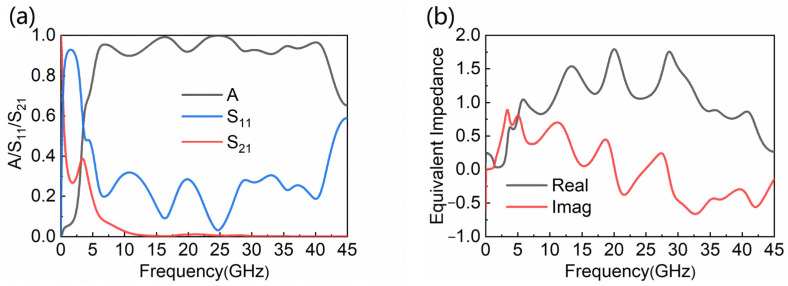
The MA’s (**a**) absorptance, *S*_11_ and *S*_21_; (**b**) equivalent impedance.

The absorptance of the designed WB absorber can be evaluated as $A=1-{\left|S_{11}\right|}^{2}-{\left|S_{21}\right|}^{2}$. Figure 2a shows that the WB absorber has a broad absorption bandwidth. In the working frequency range of 5.7–41.6 GHz, the MA can absorb over 90% of EM waves. At 16.3 GHz and 24.7 GHz, the absorptance can even reach 99.2% and 99.9%. Because the structure of the absorber is symmetrical about the *x*-*y* plane, the forward and backward absorptance are consistent, and only the forward absorption is discussed in this paper.

Impedance matching theory is introduced to ulteriorly explain the absorption mechanism of EM waves, and the normalized impedance of the WB absorber is reckoned with by using the formula expressed as [34]:

(2) $$Z=\sqrt{\frac{(1+S_{11}{}^{2})-S_{21}{}^{2}}{(1-S_{11}{}^{2})-S_{21}{}^{2}}}$$

The real and imaginary part of the equivalent impedance are around 1 and 0 at the effective frequency band in Figure 2b, which indicate that the free space is matched with the WB absorber.

**Figure 3:**
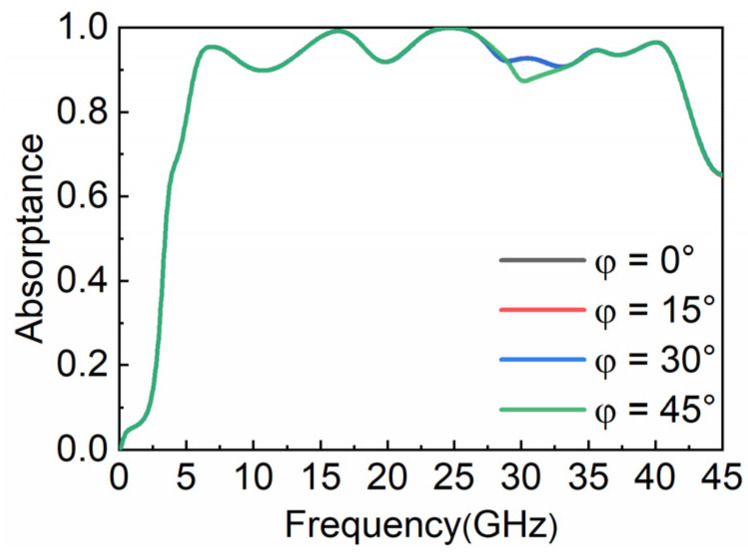
Absorptance at different polarization angles.

The polarization characteristic of the proposed MA is illustrated in Figure 3. We simulate the absorptance at vertical incidence with different polarization angles. When the polarization angle increases in the range of 0–45°, the absorptive curves of the water-based MA coincide completely, showing that the designed absorber has good polarization insensitivity, because it has a C4 structure.

**Figure 4:**
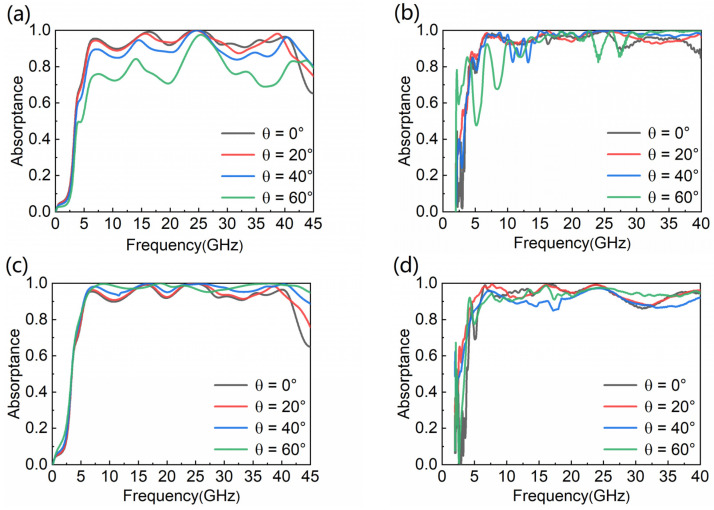
Absorptance of simulation and experiment at different angles of incidence: (**a**,**b**) TE mode; (**c**,**d**) TM mode.

To further clarify the absorptive performance of the MA, we simulate the absorptance of it in different polarization modes and at different oblique incident angles. When the incident angle gradually changes in 0–60°, and the EM wave is in TE polarization mode, the absorber can keep absorptance above 0.8 between 0° and 40°, and the bandwidth is unchanged in Figure 4a. Even when the angle of incidence reaches 60°, the absorptance can still be above 0.7 and the highest absorptance can reach 0.9. As displayed in Figure 4b, experimental results are basically in agreement with the simulations. Even at 60°, the experimental absorptance is higher than the simulated absorptance. In Figure 4c, when the angle of incidence gradually increases from 0° to 60° and the EM wave is in TM polarization mode, the absorber still can keep the absorptance above 0.9, and the bandwidth increases with the angle of oblique incidence increasing from 0° to 60°. As displayed in Figure 4d, the experimental results are well consistent with the simulations. This shows that the absorptive performance of the designed MA in the TM polarization mode is greater compared with the TE polarization mode. Additionally, the above proves that the proposed MA has great characteristics of the wide incident angle, whether in TE mode or TM mode.

**Figure 5:**
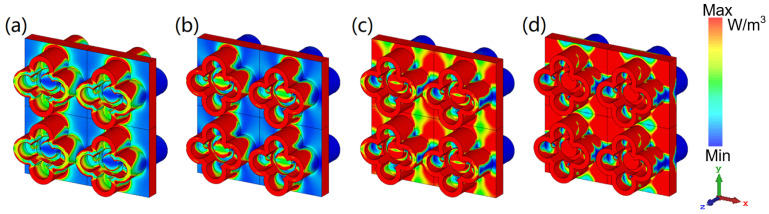
The power loss density of water layer at (**a**) 6.86 GHz; (**b**) 16.34 GHz; (**c**) 24.72 GHz; (**d**) 40 GHz.

Moreover, we monitor the power loss density and energy density of the electric and magnetic field of the water layer at four resonance points to better explain the internal loss of the MA. The all-dielectric water-based bi-directional MA is completely symmetrical about the *x*-*y* plane, so we only discuss the distribution of power loss density when the EM wave is vertically incident along the −*z* direction. Because some previous water-based MAs have proved that the power loss is mostly accumulated in the water layer of the WB absorber [43], we only monitor the power loss density of the water layer here. Whether it is low frequency or high frequency, the water layer between the two flower-shaped structures with mirror symmetry loses most of the energy at these four resonance points. It can be found from Figure 5a that the flower-shaped structure on the back has energy loss at 6.86 GHz. Therefore, the absorber is designed to be relatively thick in order to consider the energy absorption of the lower frequency part. From Figure 5b to Figure 5d, it is indicated that with the increase in frequency, the flower-shaped structure plays an increasingly significant role in energy loss in the designed MA.

**Figure 6:**
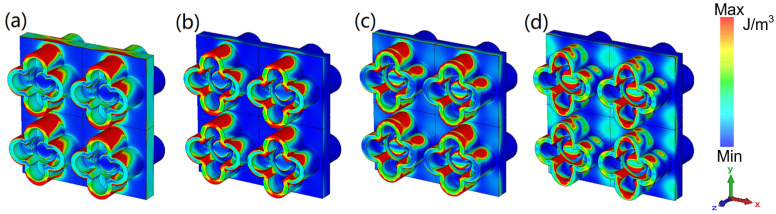
The electric field energy density of water layer at (**a**) 6.86 GHz; (**b**) 16.34 GHz; (**c**) 24.72 GHz; (**d**) 40 GHz.

**Figure 7:**
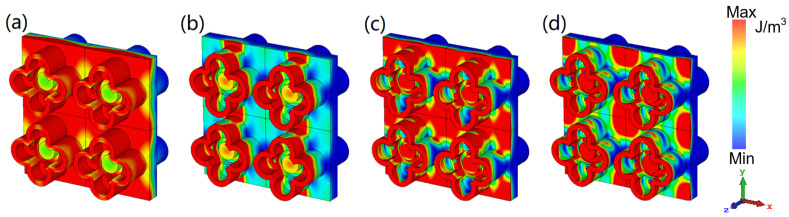
The magnetic field energy density of water layer at (**a**) 6.86 GHz; (**b**) 16.34 GHz; (**c**) 24.72 GHz; (**d**) 40 GHz.

Figure 6 and Figure 7 display the energy distribution of the electric field and magnetic field, respectively. At the four resonant points, the majority of the electric field energy is distributed in the petals’ water layer, extending toward the full water layer in the middle. Only the 6.86 GHz EM wave has the strongest penetration ability, so that part of the electric field energy is distributed in the petal water layer in the −*z* direction. For the magnetic field energy density, its distributions have a similar rule, but under the same scale, the magnetic field energy is far greater than the electric field energy, which shows that our absorber is dominated by magnetic resonance. This explains the greater angular robustness of the TM polarization mode compared to the TE mode.

**Figure 8:**
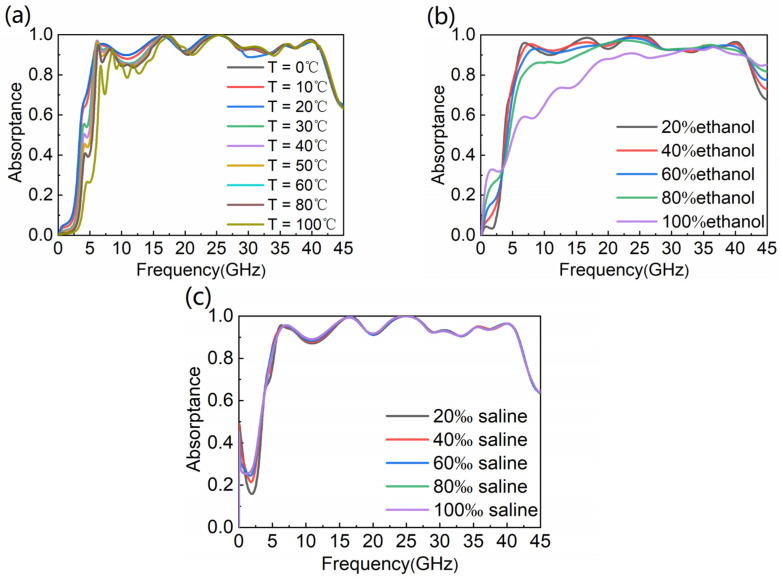
Absorptance with different (**a**) temperature; (**b**) ethanol concentrations; (**c**) salt concentrations.

Because temperature has an influence on the dielectric constant of water, it is essential to consider the absorptive performance of the WB absorber when it is injected with water at different temperatures. As seen in Figure 8a, with the temperature increasing from 0 °C to 100 °C, the absorptance of the absorber changes only slightly. When the temperature is below 80 °C, the absorber has good thermal stability in the working frequency range. In Figure 8b,c, we also simulate the absorptance of ethanol and saline with different concentrations, which shows that our water-based absorber has high tolerance to liquid, and further expands the application scenario of the absorber.

**Figure 9:**
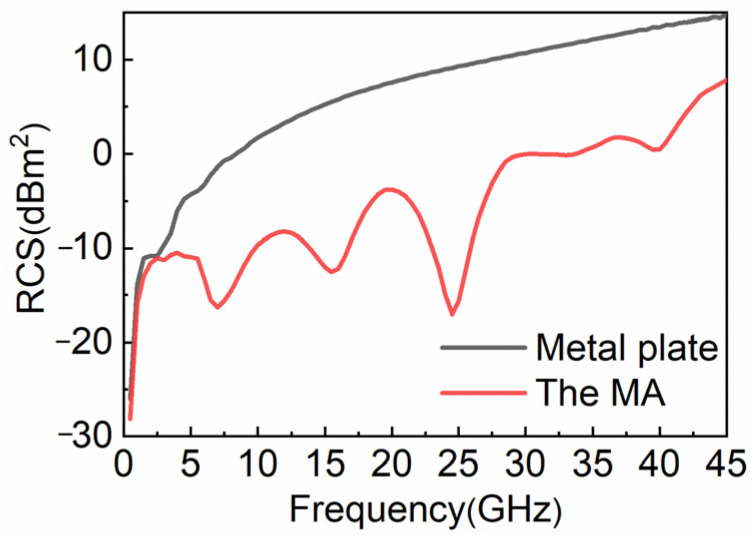
Radar cross-section of absorber and metal plate.

**Figure 10:**
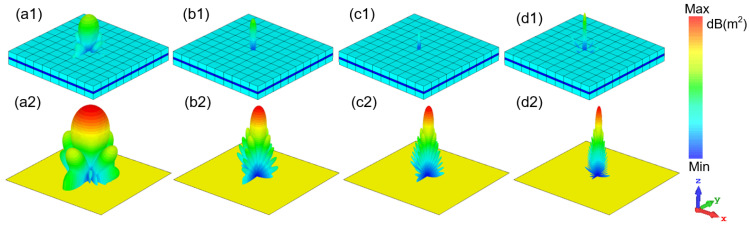
Full wave simulated scattering modes of the absorber and the metal plate at (**a1**,**a2**) 6.86 GHz; (**b1**,**b2**) 16.34 GHz; (**c1**,**c2**) 24.72 GHz; (**d1**,**d2**) 40 GHz.

In order to focus on the application of the absorber, we simulate the radar cross-section (RCS) of the absorber with 10 × 10 units and the metal plate with the same area, and we monitor the scattering modes at four resonance points. As seen in Figure 9, when the absorber compares to the metal plate, the RCS reduction effect of the MA is better than that of metal plate. The reduction effect is particularly obvious in 5.7–41.6 GHz, and the maximum reduction reaches 26 dB. In Figure 10, we compare the scattering amplitudes of the absorber and the metal plate at four resonance points. Comparing the scattering modes of the absorber and metal plate at the same frequency point, the water-based MA designed by us significantly reduces the scattering amplitude in all directions in space, especially in the incident direction. Moreover, the higher the absorptance, the better the reduction effect of RCS.

## 4. Experiment Verification

**Figure 11:**
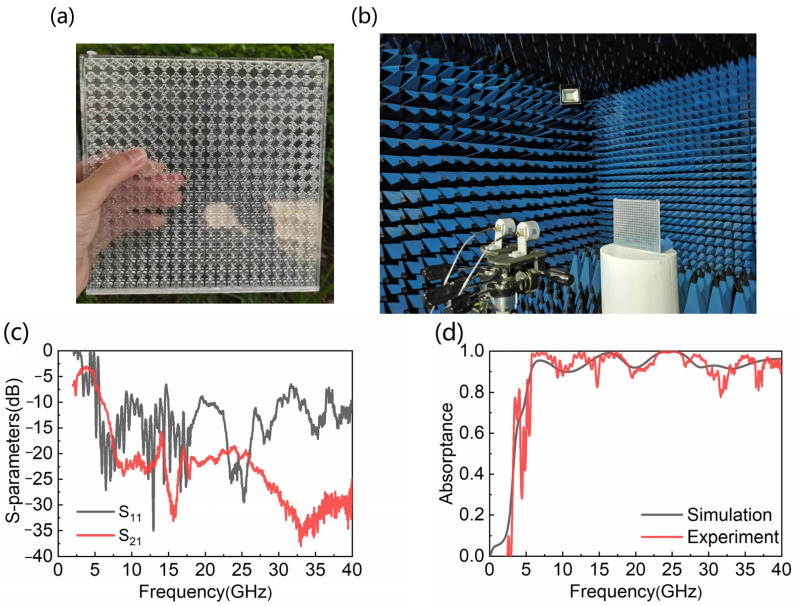
(**a**) Experimental sample; (**b**) experimental environment; (**c**) measured *S*_11_ and *S*_21_; (**d**) absorptance of simulation and experiment.

In the experiment, 3D printing technology is applied to fabricate the bi-directional water-based absorber. The transparent resin parts of the absorber are printed into two parts, and then bonded together to form a closed container. We print a 20 × 20 unit absorber sample, covering a total area of 206 × 206 × 13.2 mm^3^ in Figure 11a. For the convenience of water injection, two holes with a diameter of 3 mm are opened above the MA. Figure 11b shows the experimental environment of the absorber, which is tested by the free space method. Two circular polarized antennas are connected to a vector network analyzer (ROHDE&SCHWARZ) by a high frequency transmission line. Circular polarized antennas can verify the polarization characteristics of the WB absorber. For verifying the absorptive performance of this MA, *S*_11_ and *S*_21_ are measured in an anechoic chamber in Figure 11c. The reasons why there is more noise in *S*_11_ are: (1) there is a small angle between the two horn antennas used to measure the reflection coefficient, which does not guarantee perfect vertical transmission and reception; (2) the simulation uses an infinite plane, while the sample is finite in size, which will produce edge effects at the edge of the sample; (3) the machining accuracy of 3D printing technology is ±0.1 mm, and the samples produced may differ slightly from the simulation model. Although there is noise in *S*_11_, the overall trend is generally consistent with the simulation results. From Figure 11d, the experimental results greatly verify the simulation results. It is firmly believed that the experimental results in the unmeasured frequency band are in good consistency with the simulation results.

The comparison between the proposed absorber and some water-based absorbers is shown in Table 2, which indicates that our absorber has wider bandwidth, bi-directional absorption, transparency and it is all-dielectric. Here, we also list the unit size and operating wavelength of the relevant literature [38,39,40,41,42] to illustrate the breakthrough of the strict requirement of the sub-wavelength through their relationship.

## 5. Conclusions

In conclusion, an all-dielectric transparent WB broadband bi-directional MA has been proposed by us. The MA is based on the mixed medium of the flower-shaped water layer and resin. The flower-shaped structures in the +*z* and −*z* directions are mirror symmetry. Simulations indicate that the EM absorptance of the WB absorber exceeds 0.9 in 5.7–41.6 GHz, and the fraction bandwidth is 151.8%. In this paper, we analyze the power loss and energy distribution of the absorber, and we apply the equivalent circuit model to better understand the absorptive mechanism. For polarization insensitivity, the absorptance of the absorber is unchanged when the polarization angle is at 0–45°. Furthermore, for oblique incidence stability, in TE mode, the absorptance is over 0.8 with the incident angle increasing at 0–40°, and in TM mode, the absorptance is over 0.9 with the incident angle increasing at 0–60°. The angular stability of the absorber is verified by experiments. As for thermal stability, the absorptance of the MA is hardly changed in the temperature range of 0–80 °C. To verify the properties of the absorber in reducing RCS, we simulate and compare the RCS reduction effect of the MA and the metal plate. Based on the above superior performance and the advantage of transparency, this absorber has great application potential in stealth window weapons and electromagnetic compatibility equipment.

## Figures and Tables

**Table 1:** The Specific Value of Geometric Parameters.

Parameter	R_1_	R_2_	R_3_	R_4_	R_5_
Value (mm)	1.3	2	0.35	1.25	2
Parameter	H_1_	H_2_	H_3_	H_4_	H_5_
Value (mm)	5.6	2	2	1	4

**Table 2:** Comparison with previous works.

Ref.	RB (%)	BW (GHz)	λ_l_–λ_h_ (mm)	Periodicity	Thickness	Uni-/Bi-Directional	Transparent	All- Dielectric
[38]	66.7/100	7.5–15/5–15	40–20/60–20	8 mm: 0.2λ_l_–0.4λ_h_ /0.13λ_l_–0.4λ_h_	7 mm: 0.18λ_l_–0.35λ_h_ /0.12λ_l_–0.35λ_h_	Uni-	No	No
[39]	114.9	6.4–23.7	46.9–12.7	12.5 mm: 0.27λ_l_–0.98λ_h_	3.7 mm: 0.08λ_l_–0.29λ_h_	Uni-	Yes	No
[40]	101.1	7.74–23.56	38.8–12.73	10 mm: 0.26λ_l_–0.79λ_h_	12.8 mm: 0.33λ_l_–1λ_h_	Uni-	No	Yes
[41]	6.9	10.45–11.20	28.7–26.8	12 mm: 0.42λ_l_–0.45λ_h_	9 mm: 0.31λ_l_–0.34λ_h_	Uni-	Yes	Yes
[42]	118.0	7.28–28.22	41.2–10.6	16 mm: 0.39λ_l_–1.51λ_h_	6.5 mm: 0.16λ_l_–0.61λ_h_	Uni-	Yes	Yes
This work	151.8	5.7–41.6	52.6–7.2	10 mm: 0.19λ_l_–1.39λ_h_	13.2 mm: 0.25λ_l_–1.83λ_h_	Bi-	Yes	Yes

## Data Availability

The data that support the findings of this study are available from the corresponding authors upon reasonable request.
